# Apolipoprotein A-II Influences Apolipoprotein E-Linked Cardiovascular Disease Risk in Women with High Levels of HDL Cholesterol and C-Reactive Protein

**DOI:** 10.1371/journal.pone.0039110

**Published:** 2012-06-18

**Authors:** James P. Corsetti, Stephan J. L. Bakker, Charles E. Sparks, Robin P. F. Dullaart

**Affiliations:** 1 Department of Pathology and Laboratory Medicine, University of Rochester School of Medicine and Dentistry, Rochester, New York, United States of America; 2 Department of Nephrology, University of Groningen and University Medical Center Groningen, Groningen, The Netherlands; 3 Department of Endocrinology, University of Groningen and University Medical Center Groningen, Groningen, The Netherlands; INRCA, Italy

## Abstract

**Background:**

In a previous report by our group, high levels of apolipoprotein E (apoE) were demonstrated to be associated with risk of incident cardiovascular disease in women with high levels of C-reactive protein (CRP) in the setting of both low (designated as HR1 subjects) and high (designated as HR2 subjects) levels of high-density lipoprotein cholesterol (HDL-C).

To assess whether apolipoprotein A-II (apoA-II) plays a role in apoE-associated risk in the two female groups.

**Methodology/Principal:**

Outcome event mapping, a graphical data exploratory tool; Cox proportional hazards multivariable regression; and curve-fitting modeling were used to examine apoA-II influence on apoE-associated risk focusing on HDL particles with apolipoprotein A-I (apoA-I) without apoA-II (LpA-I) and HDL particles with both apoA-I and apoA-II (LpA-I:A-II). Results of outcome mappings as a function of apoE levels and the ratio of apoA-II to apoA-I revealed within each of the two populations, a high-risk subgroup characterized in each situation by high levels of apoE and additionally: in HR1, by a low value of the apoA-II/apoA-I ratio; and in HR2, by a moderate value of the apoA-II/apoA-I ratio. Furthermore, derived estimates of LpA-I and LpA-I:A-II levels revealed for high-risk versus remaining subjects: in HR1, higher levels of LpA-I and lower levels of LpA-I:A-II; and in HR2 the reverse, lower levels of LpA-I and higher levels of LpA-I:A-II. Results of multivariable risk modeling as a function of LpA-I and LpA-I:A-II (dichotomized as highest quartile versus combined three lower quartiles) revealed association of risk only for high levels of LpA-I:A-II in the HR2 subgroup (hazard ratio 5.31, 95% CI 1.12–25.17, p = 0.036). Furthermore, high LpA-I:A-II levels interacted with high apoE levels in establishing subgroup risk.

**Conclusions/Significance:**

We conclude that apoA-II plays a significant role in apoE-associated risk of incident CVD in women with high levels of HDL-C and CRP.

## Introduction

There is growing interest in the notion that functional properties of high-density lipoprotein (HDL) [1]–[3] are important factors contributing to protection against cardiovascular disease (CVD) risk in addition to HDL quantity. However, evidence is also accumulating to suggest that HDL athero-protective functionality may be degraded in the setting of inflammation and oxidative stress; and moreover, that such conditions may actually result in dysfunctional transformation of HDL from anti-atherogenic to pro-atherogenic [4]–[8]. To investigate this notion in human populations, we have been studying incident and recurrent CVD risk in individuals with concurrently high levels of C-reactive protein (CRP), reflective of inflammatory status, and HDL cholesterol (HDL-C). Subjects with high HDL-C levels were chosen for study instead of subjects with low HDL-C levels to avoid potential confounding of results given the well-known association of low HDL-C with risk on its own. Also in regard to the choice of high HDL-C levels, it should be noted that CVD risk associations have been reported in multiple previous studies [9]–[20]. Using this approach we have identified increased risk of incident [21]–[23] as well as recurrent [24], [25] CVD risk in populations with concurrently high levels of HDL-C and CRP.

**Figure 1:**
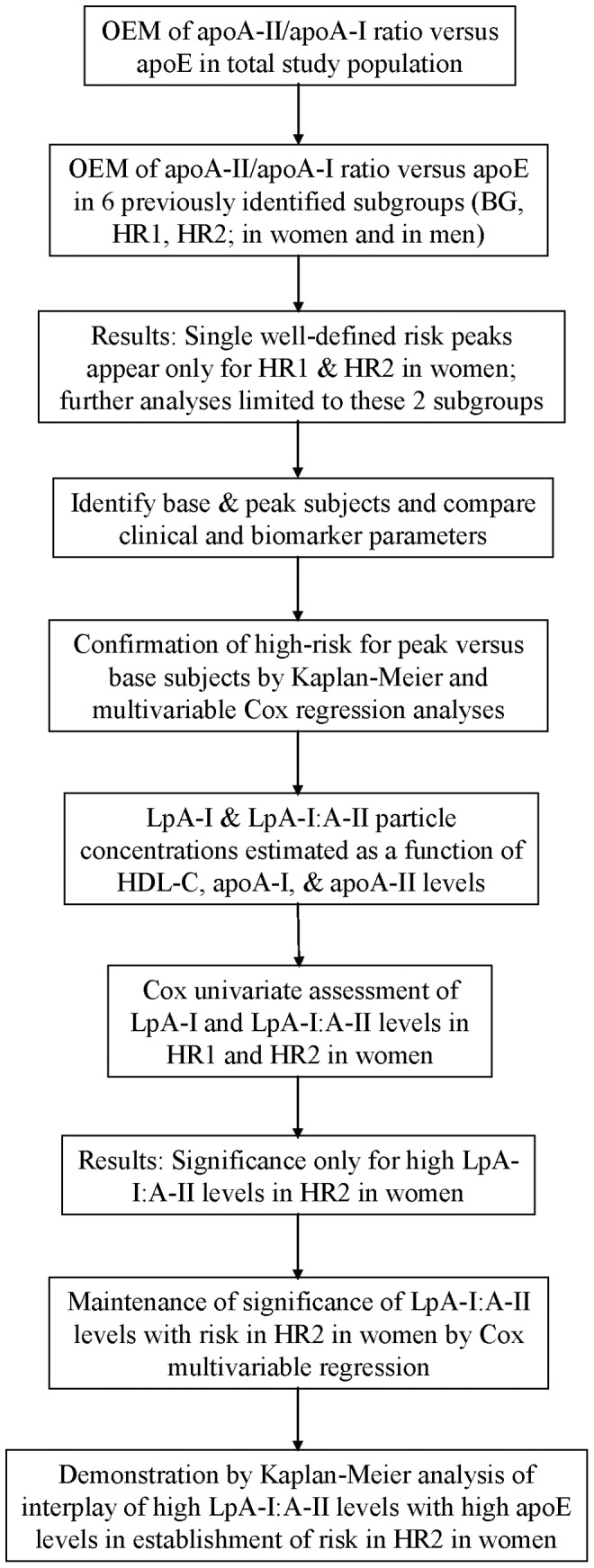
Flow diagram of study design.

**Figure 2:**
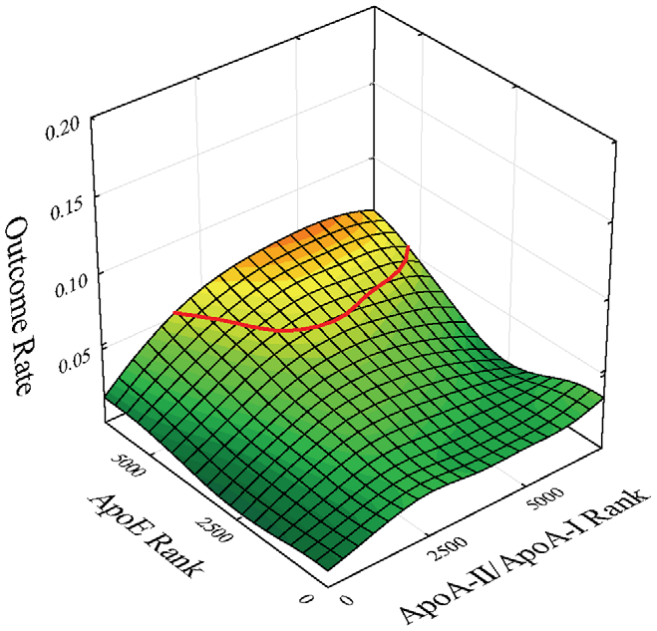
Outcome event map as a function of apoA-II/apoA-I ratio and apoE ranks in the total study population of women and men with iso-contour line of risk at 5.3% superimposed.

As anti-atherogenic properties of HDL are thought to derive from multiple HDL particle constituents including [7] apolipoproteins (apoA-I, apoA-II, and apoE), enzymes (paraoxonase-1, lecithin-cholesterol acyltransferase (LCAT), and glutathione peroxidase), and lipid transfer proteins (cholesteryl ester transfer protein (CETP) and phospholipid transfer protein (PLTP)), we recently studied and reported findings focusing on apoE in this context. Results demonstrated risk of incident CVD in association with high apoE levels in women with concurrently high levels of HDL-C and CRP [23]. In the current work, we sought to extend these studies in the same population by investigating a potential relationship in the context of risk between apoE and apoA-II, another major constituent of HDL.

ApoA-II [26]–[30] occurs in plasma as a dimer of two 77-amino acid chains linked by a disulfide bridge; and after apoA-I, it is the second major protein component of HDL accounting for approximately 20% of HDL total protein. In terms of lipoprotein physiology, the role of apoA-II has not been fully characterized; however, it is thought to play an important role in triglyceride metabolism both from animal and human studies [26], [27], [30]. In this regard, recent findings attribute to apoA-II, inhibitory effects on lipoprotein lipase-mediated hydrolysis of triglyceride-rich particles [31], [32]; however, additional associations of apoA-II have been reported for a variety of protein factors including hepatic lipase (HL), lipoprotein lipase (LPL), endothelial lipase, CETP, PLTP, and LCAT [26], [27], [33]. For apoA-II and atherosclerotic risk, the situation is again not fully characterized as apoA-II has been associated with both increased and decreased risk [33]–[35].

**Figure 3:**
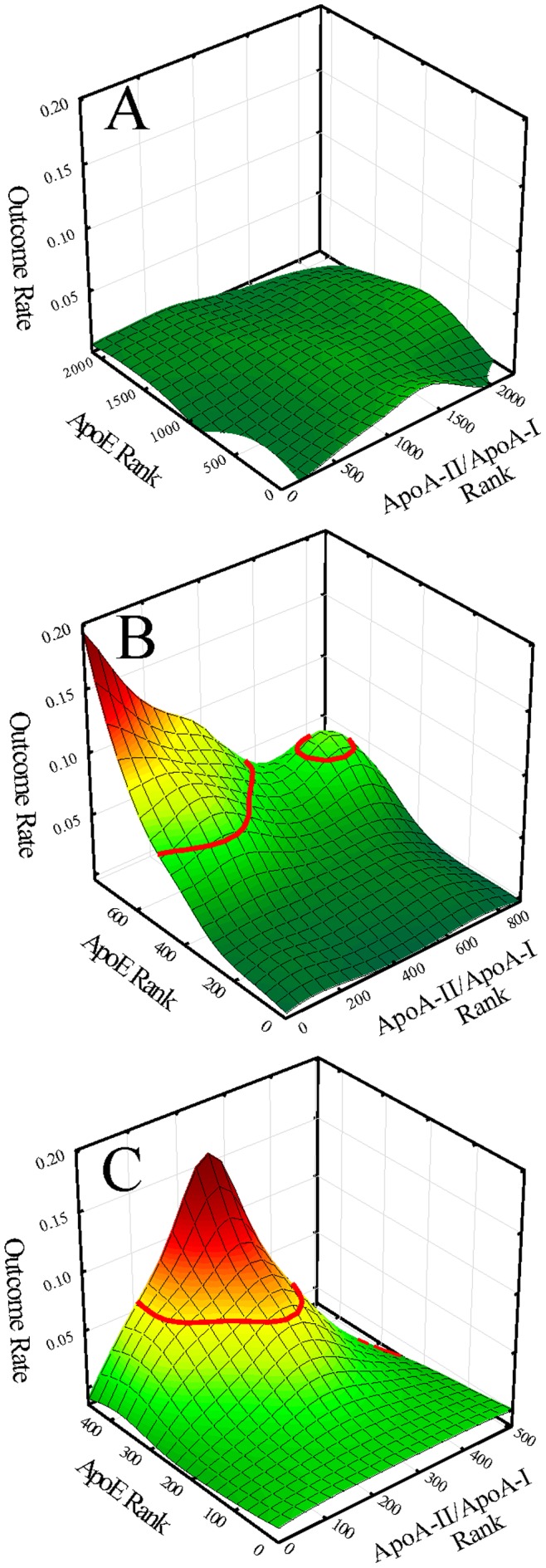
Outcome event maps as a function of apoA-II/apoA-I ratio and apoE ranks in women for: A. the background subgroup (BG); B. the low HDL-C/high CRP subgroup (HR1) with iso-contour line of risk at 5.3% superimposed; and C. the high HDL-C/high CRP subgroup (HR2) with iso-contour line of risk at 5.3% superimposed.

**Figure 4:**
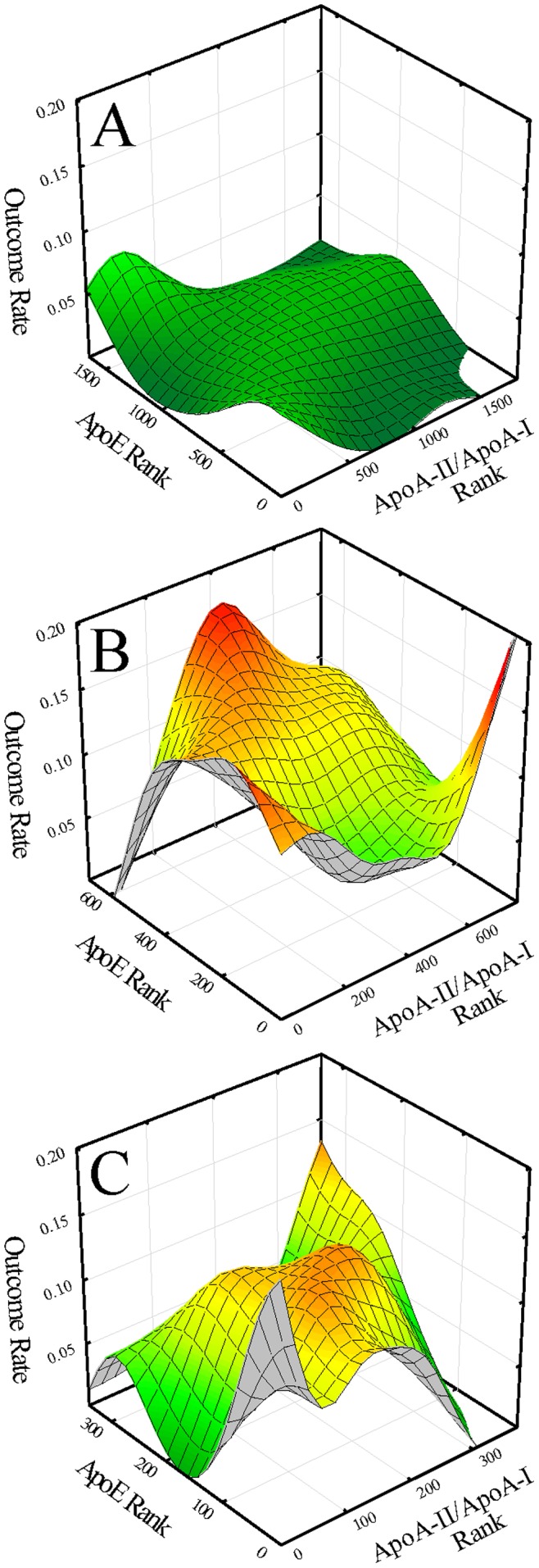
Outcome event maps as a function of apoA-II/apoA-I ratio and apoE ranks in men for: A. the background subgroup (BG); B. the low HDL-C/high CRP subgroup (HR1); and C. the high HDL-C/high CRP subgroup (HR2).

To extend our previous findings of high apoE-associated risk in women with concurrently high levels of HDL-C and CRP [23] in terms of a potential relationship with apoA-I and apoA-II, we developed a modeling approach utilizing observed levels of HDL-C, apoA-I, and apoA-II for the estimation of concentrations of the two major HDL particle subclasses [26], [27], LpA-I (containing apoA-I but not apoA-II) and LpA-I:A-II (containing apoA-I and apoA-II). With resultant estimated values for LpA-I and LpA-I:A-II particle concentrations, multivariable risk models were constructed. Results demonstrated risk association for high levels of LpA-I:A-II. Additionally, there was interaction of LpA-I:A-II with apoE in establishment of risk.

## Materials and Methods

### Ethics Statement

The PREVEND (Prevention[of Renal and Vascular Endstage Disease) study was approved by the Medical Ethics Committee of the University of Groningen, the Netherlands; written informed consent was obtained from all subjects. Detailed descriptions of definitions and data acquisition techniques were reported previously [21]–[23], [36]–[38].

**Table 1:** Clinical and laboratory characterization (mean±SD) and comparisons between base subjects and peak subjects in the female low HDL-C/high CRP high-risk subgroup (HR1).

Parameter	Total Population (N = 879)	Base (N = 705)	Peak (N = 174)	p-value
Clinical				
CVD Outcomes %(N)	3.53(31)	2.13(15)	9.20(16)	<0.0001
Age (Years)	50.1±12.3	48.5±12.1	56.4±11.3	<0.0001
BMI (kg/m^2^)	28.2±5.0	28.0±5.0	29.0±4.6	0.005
Hypertension (%)	49.0	43.9	69.5	<0.0001
Metabolic Syndrome (%)	46.3	41.3	66.1	<0.0001
Alcohol Use (%)	12.5	13.2	9.3	0.16
Smoking Status (%)				0.57
Never	31.9	31.6	33.3	
Former	28.4	27.9	30.5	
Current	39.7	40.5	36.2	
Biomarkers				
HDL-C (mmol/L)	1.07±0.16	1.07±0.16	1.08±0.14	0.72
CRP (mg/L)	3.16±2.24	3.13±2.23	3.29±2.29	0.36
apoE (g/L)	0.0422±0.0180	0.0380±0.0160	0.0569±0.0170	<0.0001
apoA-I (g/L)	1.28±0.20	1.26±0.20	1.37±0.18	<0.0001
apoA-II (g/L)	0.329±0.057	0.332±0.059	0.316±0.044	0.0001
apoA-II/apoA-I (Wt ratio)	0.258±0.034	0.265±0.034	0.231±0.018	<0.0001
HDL-C/apoA-I (mmol/g)	0.853±0.228	0.867±0.237	0.800±0.177	<0.0001
Cholesterol (mmol/L)	5.87±1.18	5.76±1.16	6.31±1.19	<0.0001
LDL-C (mmol/L)	4.00±1.04	3.93±1.00	4.27±1.16	0.0016
Triglycerides (mmol/L)	1.74±1.03	1.66±1.01	2.07±1.05	<0.0001
apoB (g/L)	1.15±0.33	1.13±0.32	1.22±0.33	0.0005

### Study Populations

The study population was drawn from PREVEND (http://www.prevend.org/) [36], a prospective longitudinal study of albuminuria in predicting cardiovascular disease [37] and renal disease [38]. All inhabitants of the city of Groningen, the Netherlands (28–75 years of age, N = 85,421) were sampled by questionnaire for demographic and cardiovascular morbidity data for the period, 1997–1998 and they were requested to supply an early morning urine specimen. Response rate was 47.8%. Study subjects included all those with urinary albumin concentrations ≥10 mg/L and a group of randomly selected subjects with urinary albumin <10 mg/L. Insulin-using diabetics and pregnant women were excluded. This gave 8592 study subjects that were equally divided by gender (median age - 48 years). Cardiovascular outcome events included cardiovascular mortality and any of the following at hospitalization: non-fatal MI (36.9%), ischemic heart disease (26.4%), percutaneous transluminal coronary angioplasty (19.2%), coronary artery bypass grafting (12.5%), and fatal MI (5.0%). Mortality data were from the municipal register. Cause of death was obtained by linking death certificate number to primary cause of death (Dutch Central Bureau of Statistics). Cardiovascular hospitalization morbidity data were from PRISMANT (Dutch national registry of hospital discharge diagnoses). Follow-up time was date of initially requested urine collection in 1997 to date of either first CVD event or study termination (31 December 2005) if no CVD event.

**Table 2:** Clinical and laboratory characterization (mean±SD) and comparisons between base subjects and peak subjects in the female high HDL-C/high CRP high-risk subgroup (HR2).

Parameter	Total Population (N = 508)	Base (N = 426)	Peak (N = 82)	p-value
Clinical				
CVD Outcomes %(N)	2.95(15)	1.64(7)	9.76(8)	<0.0001
Age (Years)	48.5±12.9	47.0±12.7	56.5±10.9	<0.0001
BMI (kg/m^2^)	27.0±4.8	26.8±4.9	27.6±4.2	0.055
Hypertension (%)	45.3	42.0	62.2	0.0008
Metabolic Syndrome (%)	12.3	10.4	22.5	0.0026
Alcohol Use (%)	17.0	16.0	22.2	0.17
Smoking Status (%)				0.11
Never	31.9	32.4	29.3	
Former	34.1	35.5	26.8	
Current	34.1	32.2	43.9	
Biomarkers				
HDL-C (mmol/L)	1.55±0.21	1.56±0.21	1.54±0.21	0.48
CRP (mg/L)	5.05±2.01	5.02±2.00	5.22±2.09	0.46
apoE (g/L)	0.0377±0.0133	0.0343±0.0114	0.0529±0.0102	<0.0001
apoA-I (g/L)	1.59±0.25	1.58±0.26	1.61±0.21	0.26
apoA-II (g/L)	0.375±0.072	0.374±0.076	0.378±0.052	0.47
apoA-II/apoA-I (Wt ratio)	0.237±0.034	0.238±0.037	0.234±0.013	0.68
HDL-C/apoA-I (mmol/g)	0.997±0.186	1.00±0.197	0.961±0.111	0.097
Cholesterol (mmol/L)	5.67±1.17	5.52±1.10	6.43±1.19	<0.0001
LDL-C (mmol/L)	3.53±1.09	3.41±1.04	4.17±1.15	<0.0001
Triglycerides (mmol/L)	1.27±0.55	1.22±0.49	1.58±0.72	<0.0001
apoB (g/L)	1.01±0.27	0.98±0.26	1.15±0.28	<0.0001

**Figure 5:**
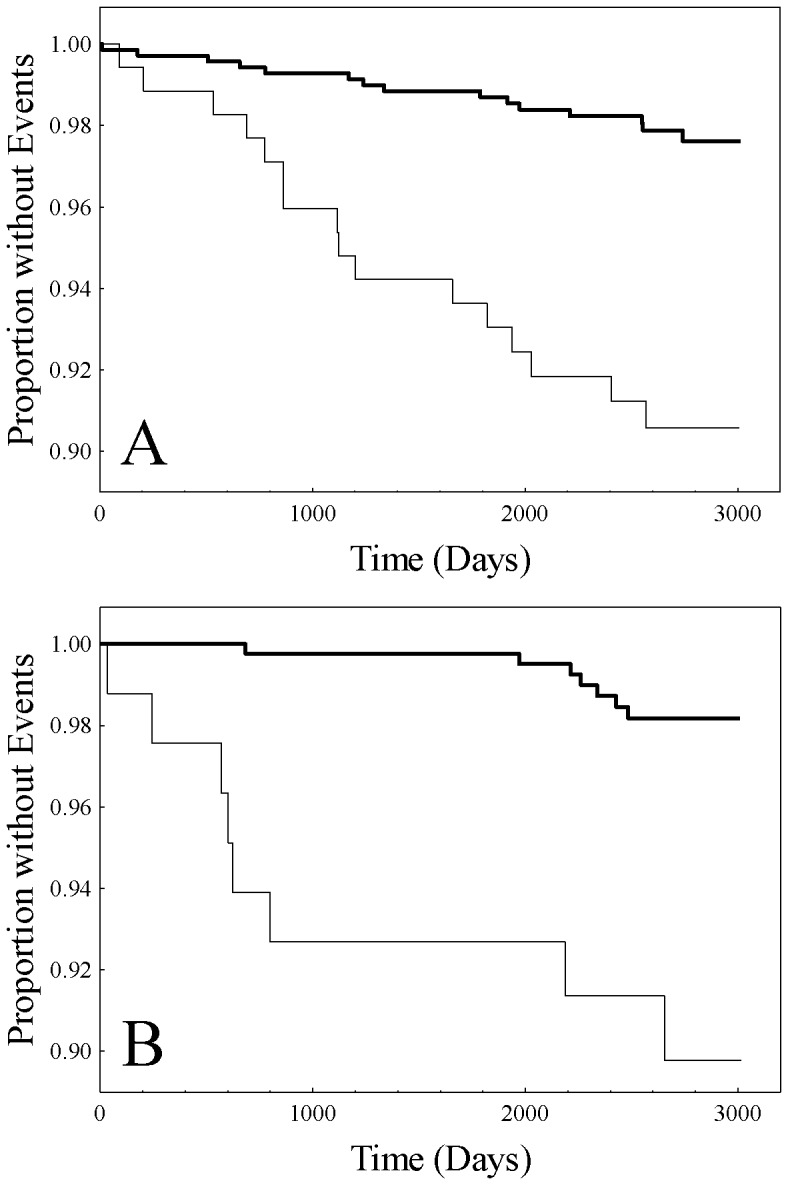
Kaplan-Meier plots for base subjects (heavy solid line) and peak subjects (light solid line) for: A. the low female HDL-C/high CRP subgroup (HR1) (p<0.0001, log-rank); and B. the female high HDL-C/high CRP subgroup (HR2) (p<0.0001, log-rank).

The six study groups of the current work consisted of sub-cohorts of female and male subjects previously identified [21]–[23] using outcome event mapping, a graphical exploratory data analysis tool [25], [39]. Subgroups were characterized as follows: female (N = 879) and male (N = 764) high-risk subgroups having concurrently low levels of HDL-C and high levels of CRP (henceforth designated HR1), female (N = 508) and male (N = 374) high-risk subgroups having concurrently high levels of HDL-C and high levels of CRP (henceforth designated HR2), and female (N = 2232) and male (N = 1813) lower-risk background subgroups of remaining subjects (henceforth designated BG). All-cause mortality rates (%) and respective % of these due to CVD for the subgroups were as follows: females, BG - 2.0%/11.1%, HR1 - 3.8%/15.2%, and HR2 - 2.8%/14.3%; and males, BG - 2.9%/13.2%, HR1 - 3.8%/27.6%, and HR2 - 10.4%/28.2%. Fractions of mortalities due to CVD did not differ among the female subgroups (p = 0.86) nor among the male subgroups (p = 0.15). Previous results of univariate modeling in the context of risk associations with apoE levels demonstrated apoE as a predictor of risk in both the low HDL-C/high CRP subgroup and high HDL-C/high CRP subgroups but only in women [23]. Initial study exclusions for PREVEND included insulin-using diabetics and pregnancy. For the current study, further exclusions were for diabetes mellitus, renal disease, previous CVD, incomplete laboratory results, and CRP levels ≥10 mg/L (to avoid confounding by inter-current illness). Median follow-up time was 7.6 years. Metabolic syndrome was assessed according to ATP III criteria.

**Table 3:** Best-fit parameters resulting from modeling of HDL-C levels as a function of apoA-I and apoA-II levels and comparison of resultant predicted values of mean HDL-C levels with observed values of mean HDL-C levels in the female low HDL-C/high CRP subgroup (HR1) and the female high HDL-C/high CRP subgroup (HR2).

	Base Subjects	Peak Subjects
HR1		
Best-fit parameters		
k1	0.0222	0.0177
k2	0.0045	0.0126
k2/k1	0.2002	0.7089
Predicted mean HDL-C (mmol/L)	1.06	1.07
Observed mean HDL-C (mmol/L)	1.07	1.08
Difference (%)	−0.93	−0.93
HR2		
Best-fit parameters		
k1	0.0257	0.0203
k2	0.0060	0.0187
k2/k1	0.2331	0.9207
Predicted mean HDL-C (mmol/L)	1.53	1.53
Observed mean HDL-C (mmol/L)	1.56	1.54
Difference (%)	−1.92	−0.65

**Table 4:** Estimates of apoA-I molecules/LpA-I particle (N), apoA-I molecules/LpA-I:A-II particle (n), and apoA-II molecules/LpA-I:A-II particle (m); and estimates of LpA-I and LpA-I:A-II particle concentrations in base and peak subjects in the female HR1 and HR2 subgroups.

	Base Subjects	Peak Subjects	p-value
HR1			
Estimated molecules/particle			
ApoA-I molecules/LpA-I particle (N)	5.07±1.73	6.14±1.33	<0.0001
ApoA-I molecules/LpA-I:A-II particle (n)	4.18±1.76	2.86±1.34	<0.0001
ApoA-II molecules/LpA-I:A-II particle (m)	4.44±2.14	4.62±1.84	0.027
Estimated particle concentrations			
LpA-I (µM)	5.05±1.00	5.88±0.84	<0.0001
LpA-I:A-II (µM)	4.31±0.77	3.93±0.54	<0.0001
HR2			
Estimated molecules/particle			
ApoA-I molecules/LpA-I particle (N)	5.15±1.68	6.30±1.35	<0.0001
ApoA-I molecules/LpA-I:A-II particle (n)	3.93±1.70	2.70±1.34	<0.0001
ApoA-II molecules/LpA-I:A-II particle (m)	5.22±1.75	3.91±1.68	<0.0001
			
Estimated particle concentrations			
LpA-I (µM)	7.46±1.41	6.53±0.85	<0.0001
LpA-I:A-II (µM)	4.14±0.86	5.35±0.74	<0.0001

### Blood Biomarkers

Biomarker analyses were performed on serum and plasma samples prepared from venous blood from subjects fasted overnight and after fifteen minutes of rest. Levels of total cholesterol, HDL-C, triglycerides, apoA1, apoB, glucose, and high sensitivity CRP were determined as described previously [21], [40]. Immunonephelometry (BNII, Dade Behring, Marburg, Germany) was used for apoE and apoA-II determinations. LDL-C levels were estimated using the Friedewald equation.

**Table 5:** Biomarker dichotomzation cut-points (Q4 versus Q1+Q2+Q3) and univariate results of Cox proportional hazards regression for LpA-I and LpA-I:A-II particle concentrations as predictors of risk as well as corresponding values for biomarker and clinical covariates in the female high HDL-C/high CRP (HR2) high-risk subgroup.

Parameter	DichotomizationCut-Off Level	Univariate HR(95% CI) p
		
LpA-I	8.25 (µM)	0.17 (0.02–1.71) 0.13
LpA-I:A-II*	5.10 (µM)	8.35 (2.21–31.46) 0.002
Biomarkers		
HDL-C	1.68 (mM)	0.46 (0.10–2.05) 0.31
CRP	6.42 (mg/L)	1.54 (0.53–4.50) 0.43
apoE*	0.0447 (g/L)	8.30 (2.20–31.28) 0.002
apoA-I	1.76 (g/L)	0.78 (0.22–2.75) 0.70
apoA-II	0.41 (g/L)	0.77 (0.22–2.72) 0.68
Cholesterol*	6.40 (mM)	4.55 (1.62–12.78) 0.004
LDL-C*	4.21 (mM)	2.61 (0.95–7.20) 0.064
Triglycerides*	1.53 (mM)	4.89 (1.74–13.75) 0.003
apoB*	1.18 (g/L)	3.46 (1.25–9.53) 0.017
Clinical Covariates		
Age (Years)*		1.08 (1.03–1.13) 0.001
BMI (kg/m^2^)*		1.10 (1.01–1.20) 0.022
Hypertension*		4.85 (1.37–17.18) 0.014
Metabolic Syndrome		1.13 (0.26–5.01) 0.87
Alcohol Use		1.22 (0.34–4.32) 0.76
Smoking Status		1.32 (0.70–2.51) 0.39

* Univariate significance at the p<0.10 level.

Adjustments to multivariable Cox models were made for biomarkers and clinical covariates demonstrating p<0.1 level of significance in Cox univariate analyses.

**Figure 6:**
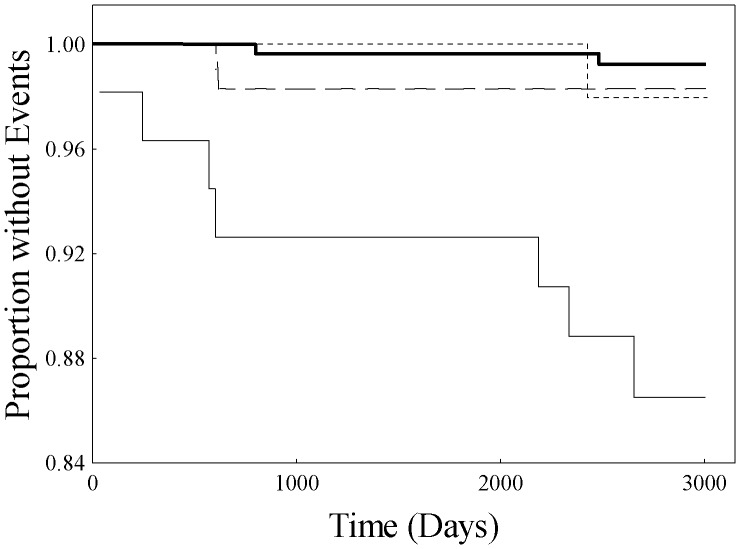
Kaplan-Meier curves in the female high-HDL-C/high CRP subgroup (HR2) as a function of combinations of dichotomized LpA-I:A-II and apoE levels as follows: light solid line - low LpA-I:A-II and low apoE, short dashes - low LpA-I:A-II and high apoE, long dashes - high LpA-I:A-II and low apoE, and heavy solid line - high LpA-I:A-II and high apoE.

### Estimation of LpA-I and LpA-I:A-II Concentrations from Observed HDL-C, apoA-I, and apoA-II Concentrations

LpA-I and LpA-I:A-II particle concentrations were estimated in four steps: 1. expressing LpA-I and LpA-I:A-II concentrations as functions of apoA-I, apoA-II, and the average number of apoA-I and apoA-II molecules per particle; 2. expressing HDL-C as a function of apoA-I, apoA-II, and the average number of apoA-I and apoA-II molecules per particle such that multiple linear regression analysis results in coefficients of the apoA-I and apoA-II terms that provide a basis for estimation of the average number of apoA-I and apoA-II molecules per particle; 3. numerical analysis for the determination of the average number of apoA-I and apoA-II molecules per LpA-I and LpA-I:A-II particles; and 4. estimation of LpA-I and LpA-I:A-II levels from observed values of apoA-I and apoA-II concentrations and the estimated values of the average number of apoA-I and apoA-II molecules per LpA-I and LpA-I:A-II particles as per step 1.

#### 1. Expressions for LpA-I and LpA-I:A-II particle concentrations

Concentrations of HDL particles containing apoA-I without apoA-II(LpA-I) and HDL particles containing apoA-I with apoA-II (LpA-I:A-II) were estimated by first assuming that the sum of apoA-I concentrations associated with each type of the two HDL particles should equal the measured apoA-I concentration as follows:
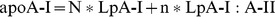
(1)where N is the average number of apoA-I molecules per LpA-I particle and n is the average number of apoA-I molecules per LpA-I:A-II particle; and second, that the apoA-II concentration should reflect the LpA-I:A-II concentration as follows:
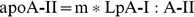
(2)where m is the average number of apoA-II molecules per LpA-I:A-II particle. An expression for LpA-I:A-II particle concentration was directly derived from equation (2) to give:
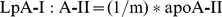
(3)while an expression for LpA-I was obtained by substituting the value of LpA-I:A-II from equation (3) into equation (1) to give:



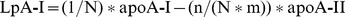
(4)This results in expressions for LpA-I and LpA-I:A-II involving measured apoA-I and apoA-II levels and three unknown parameters (N, n, and m).

#### 2. Estimation of HDL-C levels as a function of apoA-I and apoA-II levels

To estimate HDL particle concentration (HDL), a mass balance equation was formulated in terms of LpA-I and LpA-I:A-II particle concentrations as follows:
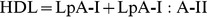
(5)where each term represents the respective particle concentrations and the assumption is that HDL exists totally either as LpA-I or LpA-I:A-II. Substitution of the values of LpA-I from equation (4) and LpA-I:A-II from equation (3) into equation (5) gives:



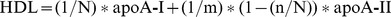
(6)However, HDL particle concentration was not measured in the study, but rather HDL-C. To re-express equation (6) in terms of HDL-C, note that:
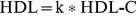
(7)where k is a linear proportionality constant relating the two. Substituting the value of HDL from equation (7) into equation (6) and solving for HDL-C gives:



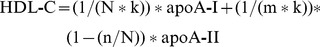
(8)The expressions for the coefficients of apoA-I and apoA-II of equation (8) involve only constants thus enabling equation (8) to be re-expressed as:
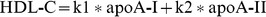
(9)


where k1 and k2 are constants defined as follow:
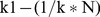
(10)

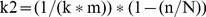
(11)


#### 3. Estimation of average number of apoA-I and apoA-II molecules per LpA-I and LpAI:A-II particle

Dividing k2 from equation (11) by k1 from equation (10) results in an expression for the ratio of k2/k1. This ratio is a function of N, n, and m but not k as follows:
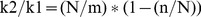
(12)


Observed HDL-C, apoA-I, and apoA-II concentrations were used with equation (9) and least-squares curve-fitting routines to generate best-fit values for the parameters, k1 and k2, and subsequently for the k2/k1 ratio (observed). In order to derive values for N, n, and M consistent with this observed ratio, a grid search was undertaken evaluating the k2/k1 ratio (predicted) resulting from judiciously chosen combinations of the three variables. Following recent work reporting that human LpA-I contains from three to seven apoA-I molecules per particle [41], N in the grid search was allowed to vary from 2 to 8 in 0.1 increments. For n and m, values were allowed to range from 1 to 8 in 0.1 increments. This procedure resulted in the sampling of 307,501 combinations. Estimates of N, n, and m were generated as mean values of the three variables for those combinations of variables that resulted in estimates of the k2/k1 ratio that were within 1% of the observed value (the percentage of combinations meeting the criterion ranged from 0.24% to 0.47% of the 307,501 sampled combinations).

#### 4. LpA-I and LpA-I:A-II concentrations

Resulting values of N, n, and m together with observed values of apoA-I and apoA-II levels were used with equations (4) and (3) to estimate values for LpA-I and LpA-I:A-II concentrations.

### Statistical Analyses

Statistica 10.0 (StatSoft, Inc., Tulsa, OK) was used for statistical, numerical, and graphical analyses. For continuous variables, differences between groups were assessed using Mann-Whitney U test; for categorical variables, differences between distributions were assessed using chi square test. Kaplan-Meier and Cox proportional hazards multivariable regression analyses were used to follow outcomes over time. Multivariable models were adjusted based on significance (p<0.10) in Cox univariate analysis for clinical covariates (age, BMI, hypertension, metabolic syndrome, ethanol use, and smoking), and blood biomarkers (HDL-C, CRP, apoA-I, apoA-II, cholesterol, LDL-C, triglycerides, apoB, and apoE). Continuous risk variables were treated as binary variables (dichotomized as highest quartile versus combined lowest three quartiles). Modeling of relationships was performed using least-squares curve-fitting routines. Significance testing of all final models was at the p<0.05 level.

Outcome event mapping [25], [39], a graphical exploratory data analysis tool, was used to identify high-risk subgroups as a function of apoE levels and the apoA-II/apoA-I ratio. Briefly, a 3-dimensional scatter plot was generated with CVD outcome on the z-axis (coded 0 for no outcome and +1 for outcome) as a function of two rank-transformed (to more evenly distribute patients over the bivariate risk domain) risk parameters (x-axis, apoE levels; y-axis, apoA-II/apoA-I ratio). A smoothing algorithm is then applied which results in a surface (outcome event map) where height above the bivariate x-y plane approximates the outcome rate. Regions of high risk are manifested as peaks while regions of low risk are manifested as valleys. Subjects contained within peaks in the mappings comprise high-risk subgroups.

## Results

### A flow Diagram to Clarify the Design of the Study is given in Figure 1

ApoA-II/apoA-I ratio and apoE-associated risk in the total study population of women and men.

Preliminary to investigating potential connections of apoE-associated risk in conjunction with apoA-I and apoA-II in HDL particles in previously identified female and male high-risk subgroups, we first studied the total study population in this regard. An outcome event map was generated for subjects of the total study population as a function of the apoA-II/apoA-I ratio and apoE level (Figure 2). The mapping shows a modest risk peak at higher levels of the apoA-II/apoA-I ratio and high levels of apoE. Cox proportional hazards regression was performed for peak versus base subjects with adjustments for clinical covariates and biomarkers demonstrating univariate significance level, p<0.1. Results of multivariable Cox regression demonstrated non-significance (p = 0.17) for peak versus base subjects in a model adjusted for gender, age, BMI, hypertension, metabolic syndrome, smoking, and levels of apoA-I, apoA-II, apoB, apoE, total cholesterol, CRP, HDL-C, and triglycerides.

### ApoA-II/apoA-I ratio and apoE-associated Risk in Subgroups

Extending investigations to previously identified high-risk subgroups in women and men of potential connections of apoE-associated risk in conjunction with apoA-I and apoA-II in HDL particles, outcome event maps in women (Figure 3) and men (Figure 4) were generated showing estimated CVD risk as a function of apoE levels and the apoA-II/apoA-I ratio for the lower-risk background subgroups (BG), the high-risk low HDL-C/high CRP subgroups (HR1), and the high-risk high HDL-C/high CRP subgroups (HR2). The plots demonstrated a well-defined single high-risk peak occurring only for HR1 women (Figure 3B, at low levels of the apoA-II/apoA-I ratio and at high levels apoE levels) and for HR2 women (Figure 3C, at an intermediate but narrow range of values for the apoA-II/apoA-I ratio and at high apoE levels). In view of the lack of focused risk associations of apoE and the apoA-II/apoA-I ratio in all three of the male and the background subgroup in women, further analyses in these subgroups were not pursued.

For the female HR1 and HR2 subgroups, higher-risk subjects were taken to be those subjects contained in the peaks of the outcome event mappings. Peak boundaries were taken to be at a base level of risk at a value of 5.3% corresponding to the onset of peaks. Figures 3B and 3C show iso-contour lines of risk at this level. On this basis, subjects contained within the high-risk peak in subsequent analyses were designated as “peak" and remaining subjects as “base" Table 1 (HR1) and Table 2 (HR2) present clinical and biomarker characterization along with statistical comparison between respective base and peak subjects. For the female HR1 subgroup (Table 1), results demonstrated for peak in comparison to base subjects older age; higher BMI; more hypertension and metabolic syndrome; higher levels of apoE, apoA-I, apoA-II, total cholesterol, LDL-C, triglycerides, and apoB; and lower apoA-II/apoA-I and HDL-C/apoA-I ratios. For the female HR2 subgroup (Table 2), results demonstrated for peak in comparison to base subjects older age, more hypertension and metabolic syndrome, and higher levels of apoE, total cholesterol, LDL-C, triglycerides, and apoB. Levels of HDL-C, apoA-I, apoA-II, and apoA-II/apoA-I ratio were not different between peak and base subjects. With regard to the apoA-II/apoA-I ratio, although mean values were not different between peak and base, the SD of the ratio for peak subjects was approximated one third the value for base subjects suggestive of a narrow range in the ratio characterizing apoE-associated risk.

To confirm the high-risk nature of peak versus base subjects, survival analysis was performed. Preliminary analyses revealed follow-up times among the BG, HR1, and HR2 subgroups to be comparable (p = 0.079, Krukal-Wallis) as well as between base and peak subjects of HR1 and HR2 (p = 0.31 and p = 0.69, respectively, Mann-Whiney-U). Results of subsequent Kaplan-Meier analysis demonstrated for the female HR1 subgroup significant difference between the curves (Figure 5A, p<0.0001, log-rank) and likewise for the female HR2 subgroup (Figure 5B, p<0.0001, log-rank). In addition, Cox proportional hazards regression was performed for peak versus base subjects with adjustments for respective Cox univariate significant clinical covariates and biomarkers for HR1 (age, BMI, hypertension, metabolic syndrome, HDL-C, cholesterol, triglycerides, and apoE) and HR2 (age, BMI, hypertension, cholesterol, LDL-C, triglycerides, apoB and apoE). Results continued to reveal significantly higher risk for peak subjects for both the HR1 subgroup (hazard ratio 2.44, 95% CI 1.07–5.55, p = 0.034) and the HR2 subgroup (hazard ratio 5.35, 95% CI 1.07–26.65, p = 0.041).

### LpA-I and LpA-I:A-II Concentrations

HDL-C concentration was modelled in terms of apoA-I and apoA-II levels as detailed in the Materials and Methods section resulting in the generation of best-fit values for the model parameters, k1 and k2. Analyses were performed separately for base and peak subjects with results given for the female HR1 and HR2 subgroups in Table 3. Predicted values of mean HDL-C levels agreed closely with corresponding observed values.

Estimates of LpA-I and LpA-I:A-II concentrations were developed as described in the Materials and Methods section. That is; values of N, n, and m resulting from grid searches based upon comparison of predicted values of the k2/k1 ratio as a function of N, n, and m combinations with the observed value of the k2/k1 ratio were incorporated along with observed apoA-I and apoA-II concentrations into equations (4) and (3) to generate estimates of LpA-I and LpA-I:A-II concentrations for base and peak subjects in the HR1 and HR2 subgroups (Table 4). For the HR1 subgroup, peak subjects versus base subjects had per LpA-I particle higher numbers of apoA-I molecules and per LpA-I:A-II particle lower numbers of apoA-I molecules and similar numbers of apoA-II molecules. For the HR2 subgroup, peak subjects versus base subjects had per LpA-I particle higher numbers of apoA-I molecules (similar to the HR1 subgroup) and per LpA-I:A-II particle lower numbers of apoA-I molecules (similar to the HR1 subgroup); however, for LpA-I:A-II particles there were fewer apoA-II molecules per particle in contrast to results for the HR1 subgroup. For the HR1 subgroup, peak versus base subjects demonstrated higher levels of LpA-I particles and lower levels of LpA-I:A-II particles. For the HR2 subgroup results were reversed; peak versus base subjects showed lower levels of LpA-I particles and higher levels of LpA-I:A-II particles.

Assessment of LpA-I and LpA-I:A-II Levels as Predictors of Risk in HR1 and HR2 Subgroups LpA-I and LpA-I:A-II levels (dichotomized as highest quartile versus combined lowest three quartiles) were first entered into univariate Cox models. For HR1, neither LpA-I (p = 0.47) nor LpA-I:A-II (p = 0.25) levels were univariate significant; for HR2, LpA-I levels were not univariate significant (p = 0.13); whereas, LpA-I:A-II levels were univariate significant (hazard ratio 8.35, 95% CI 2.21–31.46, p = 0.0017). Following the finding of univariate significance only for LPA-I:A-II levels in HR2, multivariable modeling was pursued only in this case. Models were adjusted for univariable significant biomarkers and clinical covariates. Table 5 gives dichotomization cut-points as well as Cox univariate results for biomarkers and clinical covariates. Models adjusted for significant biomarker and clinical covariates (age, BMI, hypertension, cholesterol, LDL-C, triglycerides, apoB and apoE) as a function of LpA-I:A-II levels revealed continued significance of LpA-I:A-II levels (hazard ratio 5.31, 95% CI 1.12–25.17, p = 0.036) as a predictor of risk in the HR2 subgroup.

### Relationship of apoA-II and apoE in the Establishment of Risk in HR2 Females

To investigate the relationship of apoA-II to apoE-associated risk in HR2, Kaplan-Meier analysis was performed as a function of the four combinations of dichotomized levels of LpA-I:A-II and apoE (Figure 6). The curve for subjects with high levels of both LpA-I:A-II and apoE is notable in demonstrating greater risk than any of the other three combinations (log-rank, p<0.0001; high LpA-I:A-II/high apoE subjects compared to combined remaining three lower-risk groups).

## Discussion

We previously reported identification of two subgroups of healthy women at high-risk for incident CVD, one with low HDL-C and high CRP levels and the other with high HDL-C and high CRP levels [21]–[23]. We believe the high CRP in each case to be indicative of an underlying potentiating relationship between inflammation and HDL particles in the establishment of risk. A major finding of the current work was that high levels of HDL particles with both apoA-I and apoA-II (LpA-I:A-II) associated with incident CVD risk in the high-risk group of women with high levels of HDL-C and CRP as determined by multivariable models adjusted for relevant clinical covariates and blood markers. In the same high-risk group, apoA-II levels were found not to be associated with risk which was suggestive that apoA-II associated risk derived not from the absolute amount of apoA-II but rather from the number of particles of apoA-II-carrying HDL. An additional important finding of the current study was that LpA-I:A-II associated risk was found to interact positively with the increased risk previously demonstrated for high levels of apoE in these women [23]. Regarding HDL particles with apoA-I but not apoA-II (LpA-I), there was no association of risk with LpA-I in the high HDL-C/high CRP women. Also, it should be noted that for women with low levels of HDL-C and high levels of CRP, neither LpA-I:A-II nor LpA-I associated with risk.

As stated above, we found risk in the female high HDL-C/high CRP subgroup associated with high levels of LpA-I:A-II particles but not with apoA-II levels. Regarding CVD risk and apoA-II in general, the situation is not clear in that although it is generally thought that there is an inverse relationship of risk with apoA-II levels, other studies indicate that apoA-II may be pro-atherogenic [33], [34]. Our lack of demonstrating apoA-II risk may thus be a reflection of sensitivity of potential apoA-II associated risk to salient features of specific populations. Regarding LpA-I:A-II and risk, there are few human studies directly assessing potential associations. One of particular relevance to the current study was an investigation of LpA-I and LpA-I:A-II regarding atherosclerotic lesions in hyperalphalipoproteinemic subjects. Results indicated less cardioprotective effects for higher LpA-I:A-II levels [42]. Additionally, a report from the Framingham Offspring Study in an investigation involving participants with and without coronary heart disease revealed slightly but significantly higher levels of LpA-I:A-II in cases versus HDL-C-matched controls [43]. On the other hand, earlier studies revealed for LpA-I:A-II levels in individuals with coronary artery disease as compared to controls, in one case lower levels [44] and in another case no difference [45].

With regard to apoA-II and lipoprotein metabolism, there have been substantial efforts directed at elucidation of possible links. Reported actions of apoA-II involve: primarily inhibitory effects on remodeling of HDL through modulation of activities of lipid transfer proteins (CETP, PLTP), enzymes (LCAT, LPL, HL, endothelial lipase), and receptors (SR-B1, cubilin, heat shock protein); efflux of cholesterol and phospholipids; and triglyceride metabolism [26], [27], [30]–[33]. Recent work potentially relevant to findings of the current study involved an investigation in human subjects of apoA-II metabolism in the setting of raised levels of HDL-C brought about by torcetrapib inhibition of CETP [33]. Results indicated delayed catabolism of and alterations in remodeling of apoA-II containing HDL particles presumably stemming from the large size of resultant particles and apoA-II mediated inhibition of phospholipases (endothelial lipase and HL). In addition to the previously mentioned effects of apoA-II on HDL remodeling, lipid efflux, and triglyceride metabolism; human apoA-II may play a role in the anti-oxidant properties of HDL although the issue remains unclear. One study reported the efficient transport by apoA-II rich HDL of two enzymes, paraoxonase 1 (PON1) and platelet activating factor acetylhydrolase (PAF-AH), that are thought to play a leading role in anti-oxidant functionality of HDL [46]. However, another study reported for human apoA-II enriched HDL, impaired protection against oxidative modification of apoB-containing lipoproteins and displacement of PON1 by apoA-II. This was thought to explain why PON1 is found mostly on LpA-I particles and why, at least in part, apoA-II-rich HDL demonstrated lack of anti-atherogenic properties [47]. It should also be noted that studies in mice involving apoA-II-enriched HDL have demonstrated pro-inflammatory transformation of HDL [48], [49]. In addition to these findings, a recent study reports novel apoA-II associated pro-inflammatory activity in the suppression of lipopolysaccharide (LPS) inhibition by LPS binding proteins [50]. The finding in the current study of LpA-I:A-II associated risk would thus be consistent with pro-inflammatory and pro-atherogenic characteristics of apoA-II as noted above.

Results of the current study indicated positive interaction of LpA-I:A-II with apoE in terms of CVD risk in the female high HDL-C/high CRP subgroup (HR2). In this regard it may be notable that apoA-II and apoE are known to form a heterodimeric complex that overall accounts for approximately 30% of plasma apoE in normolipidemic subjects; and furthermore, it has been reported that the complex on HDL demonstrates reduced binding affinity for the LDL receptor [51], [52]. Thus, it is tempting to speculate that the conditions of high-risk in the HR2 subgroup (high LpA-I:A-II and apoE levels) could result, in part, through formation of the apoE/apoA-II complex subsequently resulting in reduced uptake of HDL-C via the LDL receptor-mediated uptake pathway for apoE-rich HDL [53]. This could increase HDL residence time in the circulation which could foster dysfunctional transformation of HDL in the inflammatory setting indicated by the high CRP defining the subgroup.

Limitations in the current study involved a number of issues. Because the central aim of the study was to examine potential risk in the female high HDL-C/high CRP subgroup as related to apoA-II levels, and as preliminary studies revealed no evidence of such risk not only for apoA-II but additionally for apoA-I; we sought instead to extend our studies to LpA-I and LpA-I:A-II particles. This necessitated an approach involving several assumptions for the estimation of these parameters. The first of these was that LpA-I and LpA-I:A-II constitute the major subclasses of HDL. This appears justified in that although HDL particles containing apoA-II without apoA-I have been reported [54], they appear to comprise a small fraction of HDL, and in general, they are not detected in the circulation [55]. Another concern is that apoA-II is known to be present in VLDL [31]; however, this occurs in the setting of low plasma HDL-C levels which was clearly not the case in the present study of the high HDL-C subgroup. The second assumption was that HDL particle concentration was in direct proportion to measured HDL-C. Although it is known that LpA-I and LpA-I:A-II particles are heterogeneous within each subclass with regard to size and density and consequently cholesterol content; on average, the assumption of direct proportionality seems reasonable. This notion is supported by results of previous studies demonstrating statistically significant linear correlation of HDL particle concentration with HDL-C levels [56]–[58]. The third assumption was that the search for the number of apoA-I and apoA-II molecules per particle based upon goodness of fit to observed values of the k2/k1 ratio would provide valid estimates. In this regard, our estimates compared well with experimentally determined values from other studies. The value of N (number A-I molecules/LpA-I particle) in the current study ranged from 5.1 to 6.3 as compared to 3.0 to 7.0 over the total size range of HDL particles as reported in a recent study [41]. Additionally, the ratio of number of A-I molecules to number of A-II molecules in LpA-I:A-II particles in the current study ranged from 0.62 to 0.94 comparing well with reported values for human HDL of 1.00 [59], [60], 1.20 [61], 1.30 [62], 1.56 [63], and 1.89 [64]. In addition to these points, there were other limitations to the study including no direct data provided relating to the primacy of high levels of HDL-C, CRP, apoE, and LpA-I:A-II as related to dysfunctional transformation of HDL in the establishment of risk. These issues should be addressed in future studies by assessment of multiple facets of HDL functionality as well as physico-chemical characterization of HDL to elucidate the nature of potential dysfunctional transformation in such populations.

In summary, we have studied a potential role for apoA-II in the risk of incident CVD risk in a previously identified subgroup of healthy women defined by high levels of HDL-C and CRP [21], [22] and for whom apoE was previously shown to be a risk factor [23]. Results of the current study demonstrated that high levels of apoA-II containing HDL particles (LpA-I:A-II) also associated with incident CVD risk using multivariable modeling adjusted for relevant clinical and biochemical covariates. In addition, LpA-I:A-II and apoE were found to interact positively in the establishment of risk. Regarding other related factors, it should be noted that neither HDL-C, nor HDL particles without apoA-II (LpA-I), nor apoA-I, nor apoA-II levels associated with risk in the subgroup. LpA-I:A-II associated risk in the absence of apoA-II associated risk was suggestive that the number of LpA-I:A-II particles and not the quantity of apoA-II was important in the establishment of such risk. We conclude that apoA-II and apoE, major apolipoprotein constituents of HDL, are important in the ongoing characterization of the nature of HDL particles with regard to pathophysiologic mechanisms responsible for high CVD risk in populations with concurrently high levels of HDL-C and CRP.
